# Impacts of Ionizing Radiation on the Different Compartments of the Tumor Microenvironment

**DOI:** 10.3389/fphar.2016.00078

**Published:** 2016-03-30

**Authors:** Natacha Leroi, François Lallemand, Philippe Coucke, Agnès Noel, Philippe Martinive

**Affiliations:** ^1^Laboratory of Tumor and Development Biology, Groupe Interdisciplinaire de Génoprotéomique Appliquée-Cancer, University of LiègeLiège, Belgium; ^2^Cyclotron Research Center, University of LiègeLiège, Belgium; ^3^Radiotherapy-Oncology Department, Centre Hospitalier Universitaire de LiègeLiège, Belgium

**Keywords:** radiotherapy, tumor microenvironment, angiogenesis, hypoxia, inflammation, cancer-associated fibroblasts, treatment combination

## Abstract

Radiotherapy (RT) is one of the most important modalities for cancer treatment. For many years, the impact of RT on cancer cells has been extensively studied. Recently, the tumor microenvironment (TME) emerged as one of the key factors in therapy resistance. RT is known to influence and modify diverse components of the TME. Hence, we intent to review data from the literature on the impact of low and high single dose, as well as fractionated RT on host cells (endothelial cells, fibroblasts, immune and inflammatory cells) and the extracellular matrix. Optimizing the schedule of RT (i.e., dose per fraction) and other treatment modalities is a current challenge. A better understanding of the cascade of events and TME remodeling following RT would be helpful to design optimal treatment combination.

## Introduction

**FIGURE 1:**
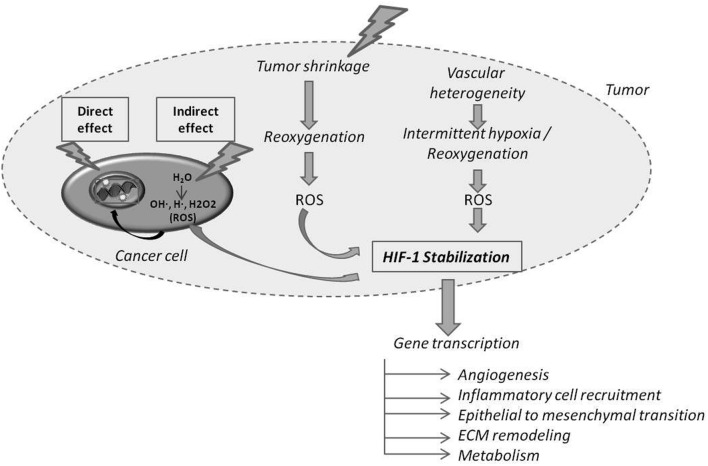
**Direct and indirect effects of radiotherapy (RT) on cancer cells and on tumor hypoxia and oxygenation.** Ionizing radiations can directly hit DNA or participate to water radiolysis generating Reactive Oxygen Species (ROS) that in turn hit DNA. The RT-induced cancer cell death of the most oxygenated cells leads to the reoxygenation of hypoxic cells. Reoxygenation favors ROS production, which in turn stabilizes Hypoxia Inducible Factor-1 (HIF-1). HIF-1 target genes are implicated in several indicated processes.

**FIGURE 2:**
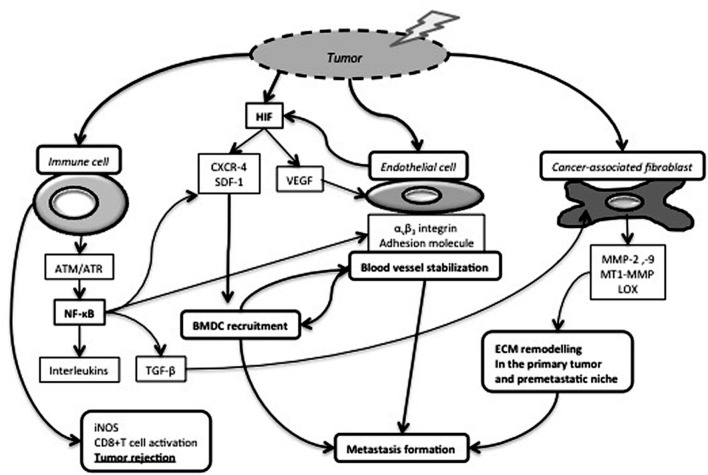
**Impact of RT on cancer-associated immune cells, endothelial cells and fibroblasts.** Tumor irradiation leads to the production and stabilization of HIF-1, which induces Vascular Endothelial Growth Factor (VEGF) production and subsequently endothelial cell proliferation and survival. Endothelial cells increase their membrane expression of α_v_β_3_ integrins and adhesion molecules. Those modifications in cell adhesion molecule expression and HIF-1-dependent CXCR-4/SDF-1 release contribute to Bone Marrow Derived Cell (BMDC) recruitment favoring in turn blood vessel stabilization and metastasis formation. RT also activates cancer-associated fibroblasts (CAF) and induces the release of extracellular matrix) (ECM) remodeling enzymes facilitating cell invasion and metastasis formation. NF-κB pathway is activated in irradiated immune cells and regulates the release of numerous cytokines, including TGF-β, an epithelial-to-mesenchymal transition inducer and a CAF activator. On the other hand, induction of inducible Nitric Oxide Synthase (iNOS) expression by tumor-associated macrophages participates to cytotoxic T cell activation and tumor rejection.

A human tumor is a complex tissue composed of malignant cells and stromal cells including endothelial cells, inflammatory cells, immune cells and fibroblasts-like cells embedded in an extracellular matrix (ECM). These cellular and extracellular components of the tumor microenvironment (TME) not only regulate different steps of cancer progression (Ribatti et al., 2006; Mandani et al., 2008; Hanahan and Weinberg, 2011), but also play a pivotal role in therapeutic efficacy (Klemm and Joyce, 2015). Radiotherapy (RT) is considered as a corner stone of cancer treatment, and more than 50% of cancer patients will experiment RT at least once during their treatment. RT can be applied alone in a curative intent or associated with chemotherapy and/or surgery performed before or after RT. High energy photons (X-rays) used in RT sparsely deposit their energy along their track. Due to physical properties of these ionizing radiations, direct events on the DNA (i.e., Double Strand Break) can be considered as rare. Most of the energy deposit occurs in water and the produced radiolysis ends up with free radical formation: Reactive Oxygen Species (ROS) and Reactive Nitrogen Species (RNS). ROS and RNS will subsequently activate several cascades and cellular processes by oxidation of molecular targets including kinases, phosphatases, cell cycle regulators, cell membrane and lipids leading to cell function dysregulation (Wu, 2006; Corre et al., 2010). These free radicals also target the DNA leading to single and double strand breaks (**Figure 1**). As RT has a non-specific effect, triggering both tumor and host cells (Valerie et al., 2007), the logical consequence is that it exerts effects beyond the simple destruction of cancer cells (Feys et al., 2015). Recent understanding that distinct stromal cell types might have tumor-promoting or tumor-suppressing capabilities (Özdemir et al., 2014) led to an even more complex picture of the tumor ecosystem and its putative impact on therapy outcome. Moreover, intriguing clinical and experimental observations reveal that the timing of surgery treatment after RT influences metastasis occurrence and patient overall survival, suggesting the implication of TME remodeling in treatment efficacy (Coucke et al., 2006; Pajonk et al., 2010; Marie-Egyptienne et al., 2013; Leroi et al., 2015). In this review, we will focus on how RT affects TME components such as the ECM, blood vessels, inflammatory and immunes cells (**Figure 2**).

## Reciprocal Dynamics Between Radiotherapy and Tumor Vessels

Tumor blood vessels are recognized as major actors in tumor development at least through an active and passive exchange of nutrients, waste and gaz (oxygen and CO_2_) between the blood stream and tumor compartment. Therefore, any modifications of these exchanges can profoundly impact the tumor phenotype. RT can affect endothelial cells directly or indirectly by inducing several cascades of events through ROS or RNS productions. RT can also indirectly impact tumor blood vessel homeostasis through the release and modification of several messengers by the tumor, which secondarily modify endothelial cell phenotype.

Garcia-Barros et al. (2003) first highlighted that microvascular radiosensitivity also influences tumor response. Membrane signaling, and especially acid sphingomyelinase/ceramide pathway, are strongly implicated in endothelial cell apoptosis after high dose RT (Corre et al., 2013). Proangiogenic factors, such as bFGF and VEGF, rapidly repress IR-induced ceramide generation, and subsequently endothelial apoptosis. Thus, combining anti-angiogenic drugs and RT would be relevant (Rao et al., 2014). On the other hand, RT leads to rapid phosphorylation of several signaling proteins (i.e., Akt and ERK) and VEGFR2, responsible of endothelial cell survival and migration (Gorski et al., 1999; Gille et al., 2001; Sofia Vala et al., 2010; Yu et al., 2012). RT also participates to endothelial activation through up-regulation of α_v_β_3_ integrin (Abdollahi et al., 2005) and adhesion molecule expression (i.e., E-selectin, P-selectin, I-CAM, V-CAM). It is also worth mentioning that RT promotes bone marrow-derived cell recruitment (**Figure 2**) (Mihaescu et al., 2007). These cells can trans-differentiate into pericytes associated to tumor blood vessels and contribute to endothelial cell radioresistance to fractionated RT (Zong et al., 2008; Lerman et al., 2010). Through vasculogenesis, CD11+ cells also participate to post-RT vasculature recovery (**Figure 2**) (Martin, 2013). RT promotes endothelial nitric oxide synthase (eNOS) expression and activation leading to NO production and finally angiogenesis and increased tumor blood flow (Sonveaux et al., 2003). Increased eNOS mRNA levels are observed after RT in human head and neck squamous cell carcinomas (Sonveaux et al., 2003). These post-RT changes of tumor vasculature are worth considering to enhance drug delivery and design treatment modalities (Sonveaux et al., 2007).

The effect on endothelial cells depends on the dose per fraction. At a clinical single dose of 2Gy, endothelial cell survival is favored through miRNA (miR-189 and miR-20a) upregulation (Wagner-Ecker et al., 2010). High doses (above 10Gy) are more likely to induce endothelial cell apoptosis and tumor vessel collapse (Park et al., 2012; Song et al., 2015). This could explain the clinical efficacy of Stereotactic Body Radiotherapy Treatments (SBRT) using high fractional dose. With intermediate doses (5-10Gy), tumor vessel normalization and dilatation are observed and associated with reduced vascular leakage and increased tumor oxygenation (Sonveaux et al., 2002; Crokart et al., 2005a).

Radiotherapy is prompt to kill the most oxygenated tumor cells thereby inducing tumor shrinkage and subsequently the perfusion and reoxygenation of initial hypoxic tumor areas (**Figure 1**) (Crokart et al., 2005a; Dewhirst et al., 2008). The reoxygenation phase following RT participates to transcriptional regulation and stabilization of HIF-1α through ROS (Kedersha et al., 1999; Moeller et al., 2004, 2005; Dewhirst et al., 2008). One direct consequence of HIF-1 and downstream target activation (i.e., PI3K/Akt, MEK/ERK and NF-κB pathways) by RT is the release of endothelial cell-derived radioprotective growth factors (VEGF and bFGF) minimizing vascular damages (Gorski et al., 1999; Kedersha et al., 1999; Moeller et al., 2004, 2005; Dewhirst et al., 2008; Sofia Vala et al., 2010; Yu et al., 2012). Interestingly, the cascade of reperfusion/reoxygenation following RT displays some similarities with intermittent hypoxia, which is a source of resistance to treatments (Martinive et al., 2006, 2009) (**Figure 1**). Moreover, RT-induced tumor cell death promotes post-irradiation angiogenesis through a caspase 3-dependent mechanism (Feng et al., 2015).

Although RT impact on tumor blood vessels is extensively studied, little is known with discrepancy results about its effect on lymphatic endothelial cells. *In vitro*, VEGF-C radiosensitizes lymphatic endothelial cells (Kesler et al., 2014). A single dose of 20Gy does not seem to alter lymphatic vessels (Pastouret et al., 2014). However, a single dose irradiation (14Gy) of murine lung tissue impairs lymphatic vasculature, progressively leading to lung fibrosis (Cui et al., 2014). In skin biopsies from irradiated breast cancer patients, similar numbers of lymphatic vessels were detected in irradiated and non-irradiated sites (Russell et al., 2015). These observations suggest a differential RT effect on blood and lymphatic endothelial cells that warrant further investigation.

## Radiotherapy and Inflammatory Signals

The link between RT and immunity is elegantly described in recent reviews (Frey et al., 2014; Barker et al., 2015; Derer et al., 2015), which highlight the importance of the chronology between RT and immunotherapy. Here, we will focus on post-RT inflammatory in the TME.

By immuno-modulatory effects, low doses RT (<1Gy) can be used as an anti-inflammatory treatment. Following low dose RT, the secretion of transforming growth factor β1 (TGF-β1), the local induction of apoptosis rather than necrosis, the decreased E-selectin expression on endothelial cell surface and the proteolytic shedding of L-selectin, altogether hamper peripheral blood mononuclear cell (PBMC) adhesion to the endothelium and subsequently inflammation. Moreover, decreased expression of interleukin-1β (IL-1β), tumor necrosis factor-α (TNF-α) and inducible nitric oxide synthase (iNOS) activity in stimulated macrophages maintain an anti-inflammatory mircoenvironment (Rodel et al., 2012). In contrary, clinical irradiation doses (≥2Gy) are known to activate inflammatory pathways in different cell types, including endothelial, immune cells and senescent fibroblasts (Mantovani et al., 2008). Furthermore, RT-induced cell death has also immunological consequences through macrophage and dendritic cell activation (Lauber et al., 2012).

Radiotherapy can initiate inflammatory cascades by two main pathways: the nuclear and cytoplasmic pathways. The first one refers to signaling events consecutive to RT-induced DNA damage. The two main effectors of DNA damage repair pathways are ataxia-telangiectasia mutated (ATM) and ATR (ATM and RAD3-related) kinases. Activated ATM can trigger NF-κB dimer activation and nuclear translocation (Wu and Miyamoto, 2007; Lavin, 2008). The cytoplasmic pathway refers to ROS-induced inactivation of phosphatases leading to the activation of Ras-Raf-MAPK and PI3K/Akt cascades. These latter also induce the expression of many genes implicated in inflammation including interleukins (IL-1α and β, IL-6, TNFα, TGF-β), adhesion molecules (I-CAM, V-CAM, E-selectin), chemokines [CCL-5, SDF1 (CXCL12)/CXCR-4] and anti-apoptotic factors (Bax and Bcl-2) (Criswell et al., 2003; Zong et al., 2008).

Bone marrow-derived cell recruitment (especially CD11b^+^ cells) following RT is largely reported in different *in vivo* models and cancer types (Vatner and Formenti, 2015). It involves mainly SDF-1/CXCR-4 (Kioi et al., 2010) and CSF-1/CSF-1R (Xu et al., 2013) pathways. The inhibition of CD11b^+^ cell recruitment through different approaches (i.e., CXCR-4 or SDF-1 inhibition) impairs tumor regrowth after single dose or fractionated RT in rat glioblastoma model and in murine prostate cancer model (Chen et al., 2013; Liu et al., 2014). CD11b^+^ cells can differentiate into endothelial cells but are also an important source of macrophages. Accordingly, SDF-1/CXCR-4 inhibition prevents macrophage infiltration and tumor regrowth after RT (Kozin et al., 2010). Macrophages are the main inflammatory cells infiltrating tumor and their role in tumor growth and dissemination depends on their polarization (M1 vs. M2) (Condeelis and Pollard, 2006). Briefly, M1 macrophages are pro-inflammatory, have a high level of iNOS production and are considered to exert anti-tumor effects. In contrast, the M2 phenotype is described as anti-inflammatory, pro-angiogenenic and pro-metastatic (Hanada et al., 2000; Mantovani et al., 2002). While the TME is recognized to affect macrophage differentiation (Weigert and Brune, 2008), the RT impact on macrophage differentiation is not well understood and is still controversial (Lambert and Paulnock, 1987; Shan et al., 2007). M2-like macrophages are preferentially attracted in hypoxic areas (Movahedi et al., 2010), in which M2 macrophage activity is fine-tuned (Laoui et al., 2014). Single high dose or fractionated doses seem to favor M2 phenotype in astrocytoma, glioma and prostate cancer models (Tsai et al., 2007; Chiang et al., 2012). On the other hand, conventional daily irradiation dose of 2Gy has been shown to convert M2-like to M1-like TAMs in melanoma xenograft model and in human pancreatic cancers. The resulting iNOS expression is responsible for vascular normalization, T cell recruitment and activation and finally tumor rejection (Klug et al., 2013). The *in vitro* exposure of THP-1 monocyte-derived macrophages to low RT doses increases IL-1β secretion in a NF-κB dependent manner, leading to an anti-inflammatory cascade (Lödermann et al., 2012). Macrophages are important NO homeostasis regulators by their differential expression of HIF-α isoforms (Takeda et al., 2010). In the presence of activated macrophages, NO is a powerful radiosensitizer for hypoxic tumor cells by inhibiting cellular respiration, which leads to oxygen sparing (De Ridder et al., 2003, 2004, 2006, 2008; Jiang et al., 2010).

NK cell mobilization following neoadjuvant RT appears crucial (Leroi et al., 2015). Indeed, TME remodeling and NK cell mobilization occurring between RT and surgery impacts the metastatic spreading. These data are in line with previous clinical data reporting that the timing of surgery following RT influences patient overall survival (Coucke et al., 2006). Interestingly, combining RT with an immunotherapy approach that triggers NK cells appears relevant, but only when RT is applied before immunotherapy (Rekers et al., 2015).

In total-body irradiation model, langerhans cells, antigen presenting cells, resist to high dose of RT (Merad et al., 2002) and induce regulatory T cell infiltration in tumors resulting in anti-tumor immunity suppression (Price et al., 2015). Moreover, in esophageal cancer, the accumulation of tumor-infiltrating regulatory T cells after neoadjuvant radiochemotherapy is associated with a worst prognosis (Vacchelli et al., 2015).

## Radiotherapy and Extracellular Matrix Remodeling

Fibroblasts are the most important producers of ECM. Normal fibroblasts are well known to resist to high radiation dose (up to 50Gy) (Tachiiri et al., 2006). Cancer-associated fibroblasts (CAF) actively contribute to cancer aggressiveness by modulating different processes (angiogenesis, inflammation and ECM remodeling) and to treatment resistance (Straussman et al., 2012; Augsten, 2014; Hirata et al., 2015). The *in vitro* crosstalk between CAF and cervical cancer cells appears to enhance cancer cell survival and proliferation following RT (Chu et al., 2014). *In vitro*, CAF isolated from lung cancer patients display similar immunosuppressive abilities following high dose RT (>5Gy) compared to non-irradiated CAF (Gorchs et al., 2015). Furthermore, the ratio between α-SMA positive (myofibroblasts) and neoplastic epithelial areas was higher after neoadjuvant RT in human rectal cancers, and was an adverse prognostic factor regarding recurrence-free survival (Verset et al., 2015). CAF presence is often viewed as a bad prognostic marker in colon (Tsujino et al., 2007), pancreatic (Erkan et al., 2008) and breast (Yamashita et al., 2012) cancers. However, a recent *in vivo* study using a murine genetic model of pancreatic ductal adenocarcinoma sheds light on an unexpected protective function of proliferating CAF (Özdemir et al., 2014). Altogether these observations suggest that different subsets of CAF can exert opposite effects on cancer progression and that RT has a propensity to induce CAF pro-tumor activity.

An intense ECM remodeling is associated with cancer progression and relies on the activity of several proteases that can be modulated by irradiation. Matrix proteolysis leads to the release of active molecules stored in the ECM, such as growth factors, angiogenic factors and active fragments of matrix components. In physiological conditions, proteolysis is tightly controlled by an appropriate balance between Matrix Metalloproteases (MMPs) and Tissue Inhibitors of Matrix Metalloproteinases (TIMPs) (Egeblad and Werb, 2002). The alteration of protease activity in tumor cells after irradiation is documented both *in vitro* and *in vivo*. MMP-2 is up-regulated following different irradiation protocols in various tumor types such as glioblastoma (Kargiotis et al., 2008), pancreatic (Qian et al., 2002), lung (Chetty et al., 2009) and colorectal cancers (Speake et al., 2005), leading to increased tumor invasion. MMP-2 inhibition before RT enhances the radiosensitivity of lung cancer cells *in vitro*. It is worth noting that proinvasive factors can be released *in vitro* from a reconstituted basement membrane (Matrigel) subjected to RT. Breast cancer cells seeded on irradiated Matrigel have increased invasion capacity with an increased expression of MT1-MMP and TIMP-2, both involved in MMP-2 activation (Paquette et al., 2007). In murine breast carcinomas, MT1-MMP blockade with a neutralizing antibody enhances the response to RT (3x6Gy) via a shift in macrophage phenotype toward anti-tumor M1-like cells associated with increased iNOS expression and tumor perfusion (Ager et al., 2015). MMP-9 expression and activity are also altered after RT in hepatocellular carcinoma cells throught the PI3K/Akt/NF-KappaB cascade (Cheng et al., 2006). In non-small cell lung carcinoma cells, after 2Gy irradiation, SDF-1/CXCR-4 pathway induces MMP expression, via Pi3K/Akt and MAPK activation, leading to increased cell invasiveness *in vitro* and *in vivo* (Gu et al., 2015).

Lysyl oxidase (LOX) is an enzyme implied in collagen and elastin fiber crosslinking, which increases ECM soluble deposition and tensile strength (Kagan and Li, 2003). The link between extracellular LOX, hypoxia and metastases is clearly demonstrated in breast cancers (Erler and Giaccia, 2006; Erler et al., 2006). LOX plays an obvious role in the premetastatic niche formation by modifying the basement membrane at the premetastatic site and thereby allowing CD11b^+^ myeloid cell recruitment (Erler et al., 2009). *In vitro*, RT increases LOX secretion in a dose-dependent manner in several tumor cell types (lung adenocarcinoma, colon carcinoma, glioma, vulva cancer, breast adenocarcinoma), which in turn promotes cancer cell invasion. Increased LOX secretion after RT was also observed *in vivo* in a lung adenocarcinoma xenograft model (Shen et al., 2014). It is worth noting that, while extracellular LOX is associated with tumor progression, intracellular LOX could be a tumor suppressor (Erler and Giaccia, 2006). Indeed, LOX propeptide inhibits prostate cancer cell growth *in vitro* and xenograft growth *in vivo* by direct interaction with DNA repair proteins leading to subsequent radio-sensitization (Bais et al., 2014). Altogether these data show that RT-induced protease release and activation varies according to the tumor type, the dose and the model.

## Conclusion

During the last decade, the initial cancer cell-centered view of tumors has greatly evolved to an integrated vision of tumor biology taking into account the key contribution of the TME. Obviously, the different compartments of TME are closely related and contribute not only to tumor progression, but also to its response to treatments. Importantly, the TME evolves over time during the different steps of cancer development and is also affected by different therapeutic modalities. Although, improvements have been achieved regarding RT delivery to the primary tumor, ionizing radiation also target non-tumor cells that influence tumor growth and metastatic dissemination. Different approaches have been proposed to overcome the radioresistance of cancer cells. The TME-mediated radioresistance is now the object of researches, which has been elegantly reviewed recently by Barker et al. (2015) and several articles pointed out the importance of treatments that modify the TME and likely radiosensitize tumor (Ansiaux et al., 2005; Crokart et al., 2005b; Frérart et al., 2008).

However, the impact of anti-cancer treatments on the TME and consequently on the tumor phenotype, response to treatment and metastases, is often neglected. Here we pointed out the impact of RT on the TME. Recent findings emphasize the interest to optimize RT (i.e., dose per fraction) and timing of surgery (Leroi et al., 2015; Surace et al., 2015) in order to prevent metastatic spreading. The future challenge in RT will be to define the most appropriate combinations between RT, and other therapeutic modalities with the optimal sequence and timing of treatments. In this context, investigation of the TME-related acquired resistance will be essential and will provide important innovative data.

## Author Contributions

NL wrote the manuscript including figures and gathered manuscript modifications from the authors. AN and PM designed and wrote the manuscript. FL contributed to figures and reviewed the manuscript. PC reviewed the manuscript.

## Conflict of Interest Statement

The authors declare that the research was conducted in the absence of any commercial or financial relationships that could be construed as a potential conflict of interest.

## References

[B1] Abdollahi A. Griggs D. W. Zieher H. Roth A. Lipson K. E. Saffrich R. (2005). Inhibition of alpha(v)beta3 integrin survival signaling enhances antiangiogenic and antitumor effects of radiotherapy. *Clin. Cancer Res.* 11 6270–6279. 10.1158/1078-0432.CCR-04-1223 16144931

[B2] Ager E. I. Kozin S. V. Kirkpatrick N. D. Seano G. Kodack D. P. Askoxylakis V. (2015). Blockade of MMP14 activity in murine breast carcinomas: implications for macrophages, vessels, and radiotherapy. *J. Natl. Cancer Instit.* 107:djv017 10.1093/jnci/djv017 PMC4402365 25710962

[B3] Ansiaux R. Baudelet C. Jordan B. F. Beghein N. Sonveaux P. De Wever J (2005). Thalidomide radiosensitizes tumors through early changes in the tumor microenvironment. *Clin. Cancer Res.* 11(2 Pt 1) 743–750. 15701864

[B4] Augsten M. (2014). Cancer-associated fibroblasts as another polarized cell type of the tumor microenvironment. *Front. Oncol.* 4:62 10.3389/fonc.2014.00062 PMC3973916 24734219

[B5] Bais M. V. Ozdener G. B. Sonenshein G. E. Trackman P. C. (2014). Effects of tumor-suppressor lysyl oxidase propeptide on prostate cancer xenograft growth and its direct interactions with DNA repair pathways. *Oncogene* 34 1928–1937. 10.1038/onc.2014.147 24882580 PMC4254378

[B6] Barker H. E. Paget J. T. Khan A. A. Harrington K. J. (2015). The tumour microenvironment after radiotherapy: mechanisms of resistance and recurrence. *Nat. Rev. Cancer* 15 409–425. 10.1038/nrc3958 26105538 PMC4896389

[B7] Chen F. H. Fu S. Y. Yang Y. C. Wang C. C. Chiang C. S. Hong J. H. (2013). Combination of vessel-targeting agents and fractionated radiation therapy: the role of the SDF-1/CXCR4 pathway. *Int. J. Radiat. Oncol. Biol. Phys.* 86 777–784. 10.1016/j.ijrobp.2013.02.036 23601898

[B8] Cheng J. C. Chou C. H. Kuo M. L. Hsieh C. Y. (2006). Radiation-enhanced hepatocellular carcinoma cell invasion with MMP-9 expression through PI3K//Akt//NF-[kappa]B signal transduction pathway. *Oncogene* 25 7009–7018. 10.1038/sj.onc.1209706 16732316

[B9] Chetty C. Bhoopathi P. Rao J. S. Lakka S. S. (2009). Inhibition of matrix metalloproteinase-2 enhances radiosensitivity by abrogating radiation-induced FoxM1-mediated G2/M arrest in A549 lung cancer cells. *Int. J. Cancer* 124 2468–2477. 10.1002/ijc.24209 19165865 PMC2663016

[B10] Chiang C. S. Fu S. Y. Wang S. C. Yu C. F. Chen F. H. Lin C. M. (2012). Irradiation promotes an m2 macrophage phenotype in tumor hypoxia. *Front. Oncol.* 2:89 10.3389/fonc.2012.00089 PMC3412458 22888475

[B11] Chu T. Y. Yang J. T. Huang T. H. Liu H. W. (2014). Crosstalk with cancer-associated fibroblasts increases the growth and radiation survival of cervical cancer cells. *Radiat. Res.* 181 540–547. 10.1667/RR13583.1 24785588

[B12] Condeelis J. Pollard J. W. (2006). Macrophages: obligate partners for tumor cell migration, invasion, and metastasis. *Cell* 124 263–266. 10.1016/j.cell.2006.01.007 16439202

[B13] Corre I. Guillonneau M. Paris F. (2013). Membrane signaling induced by high doses of ionizing radiation in the endothelial compartment. Relevance in radiation toxicity. *Int. J. Mol. Sci.* 14 22678–22696. 10.3390/ijms141122678 24252908 PMC3856084

[B14] Corre I. Niaudet C. Paris F. (2010). Plasma membrane signaling induced by ionizing radiation. *Mutat. Res.* 704 61–67. 10.1016/j.mrrev.2010.01.014 20117234

[B15] Coucke P. A. Notter M. Matter M. Fasolini F. Calmes J. M. Schlumpf R. (2006). Effect of timing of surgery on survival after preoperative hyperfractionated accelerated radiotherapy (HART) for locally advanced rectal cancer (LARC): is it a matter of days? *Acta Oncol.* 45 1086–1093. 10.1080/02841860600891317 17118844

[B16] Criswell T. Leskov K. Miyamoto S. Luo G. Boothman D. A. (2003). Transcription factors activated in mammalian cells after clinically relevant doses of ionizing radiation. *Oncogene* 22 5813–5827. 10.1038/sj.onc.1206680 12947388

[B17] Crokart N. Jordan B. F. Baudelet C. Ansiaux R. Sonveaux P. Grégoire V. (2005a). Early reoxygenation in tumors after irradiation: determining factors and consequences for radiotherapy regimens using daily multiple fractions. *Int. J. Radiat. Oncol. Biol. Phys.* 63 901–910. 10.1016/j.ijrobp.2005.02.038 16199320

[B18] Crokart N. Radermacher K. Jordan B. F. Baudelet C. Cron G. O. Grégoire V. (2005b). Tumor radiosensitization by antiinflammatory drugs: evidence for a new mechanism involving the oxygen effect. *Cancer Res.* 65 7911–7916. 16140962 10.1158/0008-5472.CAN-05-1288

[B19] Cui Y. Wilder J. Rietz C. Gigliotti A. Tang X. Shi Y. (2014). Radiation-induced impairment in lung lymphatic vasculature. *Lymphatic Res. Biol.* 12 238–250. 10.1089/lrb.2014.0012 PMC4267131 25412238

[B20] De Ridder M. Jiang H. Van Esch G. Law K. Monsaert C. Van den Berge D. L. (2008). IFN-γ+ CD8^+^ T lymphocytes: possible link between immune and radiation responses in tumor-relevant hypoxia. *Int. J. Radiat. Oncol. Biol. Phys.* 71 647–651. 10.1016/j.ijrobp.2008.03.014 18514774

[B21] De Ridder M. Verovski V. N. Chiavaroli C. Van den Berge D. L. Monsaert C. Law K. (2006). The radiosensitizing effect of immunoadjuvant OM-174 requires cooperation between immune and tumor cells through interferon-gamma and inducible nitric oxide synthase. *Int. J. Radiat. Oncol. Biol. Phys.* 66 1473–1480. 10.1016/j.ijrobp.2006.07.1381 17056198

[B22] De Ridder M. Verovski V. N. Darville M. I. Van Den Berge D. L. Monsaert C. Eizirik D. L. (2004). Macrophages enhance the radiosensitizing activity of lipid A: a novel role for immune cells in tumor cell radioresponse. *Int. J. Radiat. Oncol. Biol. Phys.* 60 598–606. 10.1016/j.ijrobp.2004.05.065 15380597

[B23] De Ridder M. Verovski V. N. Van den Berge D. L. Sermeus A. B. Monsaert C. Wauters N. (2003). Lipid a radiosensitizes hypoxic EMT-6 tumor cells: role of the NF-κB signaling pathway. *Int. J. Radiat. Oncol. Biol. Phys.* 57 779–786. 10.1016/S0360-3016(03)00662-X 14529784

[B24] Derer A. Frey B. Fietkau R. Gaip U. S. (2015). Immune-modulating properties of ionizing radiation: rationale for the treatment of cancer by combination radiotherapy and immune checkpoint inhibitors. *Cancer Immunol. Immunother.* 10.1007/s00262-015-1771-8 [Epub ahead of print]. PMC11028616 26590829

[B25] Dewhirst M. W. Cao Y. Moeller B. (2008). Cycling hypoxia and free radicals regulate angiogenesis and radiotherapy response. *Nat. Rev. Cancer* 8 425–437. 10.1038/nrc2397 18500244 PMC3943205

[B26] Egeblad M. Werb Z. (2002). New functions for the matrix metalloproteinases in cancer progression. *Nat. Rev. Cancer* 2 161–174. 10.1038/nrc745 11990853

[B27] Erkan M. Michalski C. W. Rieder S. Reiser-Erkan C. Abiatari I. Kolb A. (2008). The activated stroma index is a novel and independent prognostic marker in pancreatic ductal adenocarcinoma. *Clin. Gastroenterol. Hepatol.* 6 1155–1161. 10.1016/j.cgh.2008.05.006 18639493

[B28] Erler J. T. Bennewith K. L. Cox T. R. Lang G. Bird D. Koong A. (2009). Hypoxia-induced lysyl oxidase is a critical mediator of bone marrow cell recruitment to form the premetastatic niche. *Cancer Cell* 15 35–44. 10.1016/j.ccr.2008.11.012 19111879 PMC3050620

[B29] Erler J. T. Bennewith K. L. Nicolau M. Dornhöfer N. Kong C. Le Q. T. (2006). Lysyl oxidase is essential for hypoxia-induced metastasis. *Nature* 440 1222–1226. 10.1038/nature04695 16642001

[B30] Erler J. T. Giaccia A. J. (2006). Lysyl oxidase mediates hypoxic control of metastasis. *Cancer Res.* 66 10238–10241. 10.1158/0008-5472.CAN-06-3197 17079439

[B31] Feng X. Tian L. Zhang Z. Yu Y. Cheng J. Gong Y. (2015). Caspase 3 in dying tumor cells mediates post-irradiation angiogenesis. *Oncotarget* 6 32353–32367. 10.18632/oncotarget.5898 26431328 PMC4741698

[B32] Feys L. Descamps B. Vanhove C. Vral A. Veldeman L. Vermeulen S. (2015). Radiation-induced lung damage promotes breast cancer lung-metastasis through CXCR4 signaling. *Oncotarget* 6 26615–26632. 10.18632/oncotarget.5666 26396176 PMC4694940

[B33] Frérart F. Sonveaux P. Rath G. Smoos A. Meqor A. Charlier N. (2008). The acidic tumor microenvironment promotes the reconversion of nitrite into nitric oxide: towards a new and safe radiosensitizing strategy. *Clin. Cancer Res.* 14 2768–2774. 10.1158/1078-0432.CCR-07-4001 18451244

[B34] Frey B. Rubner Y. Kulzer L. Werthmöller N. Weiss E. M. Fietkau R. (2014). Antitumor immune responses induced by ionizing irradiation and further immune stimulation. *Cancer Immunol. Immunother.* 63 29–36. 10.1007/s00262-013-1474-y 24052136 PMC11028436

[B35] Garcia-Barros M. Paris F. Cordon-Cardo C. Lyden D. Rafii S. Haimovitz-Friedman A. (2003). Tumor response to radiotherapy regulated by endothelial cell apoptosis. *Science* 300 1155–1159. 10.1126/science.1082504 12750523

[B36] Gille H. Kowalski J. Li B. LeCouter J. Moffat B. Zioncheck T. F. (2001). Analysis of biological effects and signaling properties of Flt-1 (VEGFR-1) and KDR (VEGFR-2). *J. Biol. Chem.* 276 3222–3230. 10.1074/jbc.M002016200 11058584

[B37] Gorchs L. Hellevik T. Bruun J. A. Camilio K. A. Al-Saad S. Stuge T. B. (2015). Cancer-associated fibroblasts from lung tumors maintain their immunosuppressive abilities after high-dose irradiation. *Front. Oncol.* 5:87 10.3389/fonc.2015.00087 PMC4429237 26029659

[B38] Gorski D. H. Beckett M. A. Jaskowiak N. T. Calvin D. P. Mauceri H. J. Salloum R. M. (1999). Blockade of the vascular endothelial growth factor stress response increases the antitumor effects of ionizing radiation. *Cancer Res.* 59 3374–3378. 10416597

[B39] Gu Q. He Y. Ji J. Yao Y. Shen W. Luo J. (2015). Hypoxia-inducible factor 1α (HIF-1α) and reactive oxygen species (ROS) mediates radiation-induced invasiveness through the SDF-1α/CXCR4 pathway in non-small cell lung carcinoma cells. *Oncotarget* 6:13 10.18632/oncotarget.3535 PMC4484427 25843954

[B40] Hanada T. Nakagawa M. Emoto A. Nomura T. Nasu N. Nomura Y. (2000). Prognostic value of tumor-associated macrophage count in human bladder cancer. *Int. J. Urol.* 7 263–269. 10.1046/j.1442-2042.2000.00190.x 10910229

[B41] Hanahan D. Weinberg R. A. (2011). Hallmarks of cancer: the next generation. *Cell* 144 646–674. 10.1016/j.cell.2011.02.013 21376230

[B42] Hirata E. Girotti M. R. Viros A. Hooper S. Spencer-Dene B. Matsuda M. (2015). Intravital imaging reveals how BRAF inhibition generates drug-tolerant microenvironments with high integrin β1/FAK Signaling. *Cancer Cell* 27 574–588. 10.1016/j.ccell.2015.03.008 25873177 PMC4402404

[B43] Jiang H. De Ridder M. Verovski V. N. Sonveaux P. Jordan B. F. Law K. (2010). Activated macrophages as a novel determinant of tumor cell radioresponse: the role of nitric oxide–mediated inhibition of cellular respiration and oxygen sparing. *Int. J. Radiat. Oncol. Biol. Phys.* 76 1520–1527. 10.1016/j.ijrobp.2009.10.047 20338478

[B44] Kagan H. M. Li W. (2003). Lysyl oxidase: properties, specificity, and biological roles inside and outside of the cell. *J. Cell. Biochem.* 88 660–672. 10.1002/jcb.10413 12577300

[B45] Kargiotis O. Chetty C. Gondi C. S. Tsung A. J. Dinh D. H. Gujrati M. (2008). Adenovirus-mediated transfer of siRNA against MMP-2 mRNA results in impaired invasion and tumor-induced angiogenesis, induces apoptosis in vitro and inhibits tumor growth in vivo in glioblastoma. *Oncogene* 27 4830–4840. 10.1038/onc.2008.122 18438431 PMC2574662

[B46] Kedersha N. L. Gupta M. Li W. Miller I. Anderson P. (1999). RNA-binding proteins Tia-1 and tiar link the phosphorylation of eif-2α to the assembly of mammalian stress granules. *J. Cell Biol.* 147 1431–1442. 10.1083/jcb.147.7.1431 10613902 PMC2174242

[B47] Kesler C. T. Kuo A. H. Wong H. K. Masuck D. J. Shah J. L. Kozak K. R. (2014). Vascular endothelial growth factor-C enhances radiosensitivity of lymphatic endothelial cells. *Angiogenesis* 17 419–427. 10.1007/s10456-013-9400-7 24201897 PMC3981926

[B48] Kioi M. Vogel H. Schultz G. Hoffman R. M. Harsh G. R. Brown J. M. (2010). Inhibition of vasculogenesis, but not angiogenesis, prevents the recurrence of glioblastoma after irradiation in mice. *J. Clin. Investig.* 120 694–705. 10.1172/JCI40283 20179352 PMC2827954

[B49] Klemm F. Joyce J. A. (2015). Microenvironmental regulation of therapeutic response in cancer. *Trends Cell Biol.* 25 198–213. 10.1016/j.tcb.2014.11.006 25540894 PMC5424264

[B50] Klug F. Prakash H. Huber P. E. Seibel T. Bender N. Halama N. (2013). Low-dose irradiation programs macrophage differentiation to an iNOS+/M1 phenotype that orchestrates effective T cell immunotherapy. *Cancer Cell* 24 589–602. 10.1016/j.ccr.2013.09.014 24209604

[B51] Kozin S. V. Kamoun W. S. Huang Y. Dawson M. R. Jain R. K. Duda D. G. (2010). Recruitment of myeloid but not endothelial precursor cells facilitates tumor regrowth after local irradiation. *Cancer Res.* 70 5679–5685. 10.1158/0008-5472.CAN-09-4446 20631066 PMC2918387

[B52] Lambert L. E. Paulnock D. M. (1987). Modulation of macrophage function by gamma-irradiation. Acquisition of the primed cell intermediate stage of the macrophage tumoricidal activation pathway. *J. Immunol.* 139 2834–2841. 3116096

[B53] Laoui D. Van Overmeire E. Di Conza G. Aldeni C. Keirsse J. Morias Y. (2014). Tumor hypoxia does not drive differentiation of tumor-associated macrophages but rather fine-tunes the M2-like macrophage population. *Cancer Res.* 74 24–30. 10.1158/0008-5472.CAN-13-1196 24220244

[B54] Lauber K. Ernst A. Orth M. Herrmann M. Belka C. (2012). Dying cell clearance and its impact on the outcome of tumor radiotherapy. *Front. Oncol.* 2:116 10.3389/fonc.2012.00116 PMC3438527 22973558

[B55] Lavin M. F. (2008). Ataxia-telangiectasia: from a rare disorder to a paradigm for cell signalling and cancer. *Nat. Rev. Mol. Cell Biol.* 9 759–769. 10.1038/nrm2514 18813293

[B56] Lerman O. Z. Greives M. R. Singh S. P. Thanik V. D. Chang C. C. Seiser N. (2010). Low-dose radiation augments vasculogenesis signaling through HIF-1-dependent and -independent SDF-1 induction. *Blood* 116 3669–3676. 10.1182/blood-2009-03-213629 20631377

[B57] Leroi N. Sounni N. E. Van Overmeire E. Blacher S. Marée R. Van Ginderachter J. (2015). The timing of surgery after neoadjuvant radiotherapy influences tumor dissemination in a preclinical model. *Oncotarget* 6 36825–36837. 10.18632/oncotarget.5931 26440148 PMC4742213

[B58] Liu S. C. Alomran R. Chernikova S. B. Lartey F. Stafford J. Jang T. (2014). Blockade of SDF-1 after irradiation inhibits tumor recurrences of autochthonous brain tumors in rats. *Neuro Oncol.* 16 21–28. 10.1093/neuonc/not149 24335554 PMC3870826

[B59] Lödermann B. Wunderlich R. Frey S. Schorn C. Stangl S. Rödel F. (2012). Low dose ionising radiation leads to a NF-kappaB dependent decreased secretion of active IL-1beta by activated macrophages with a discontinuous dose-dependency. *Int. J. Radiat. Biol.* 88 727–734. 10.3109/09553002.2012.689464 22545750

[B60] Mandani I. De Neve W. Mareel M. (2008). Does ionizing radiation stimulate cancer invasion and metastasis? *Bull Cancer* 95 292–300. 10.1684/bdc.2008.0598 18390409

[B61] Mantovani A. Allavena P. Sica A. Balkwi F. (2008). Cancer-related inflammation. *Nature* 454 436–444. 10.1038/nature07205 18650914

[B62] Mantovani A. Sozzani S. Locati M. Allavena P. Sica A. (2002). Macrophage polarization: tumor-associated macrophages as a paradigm for polarized M2 mononuclear phagocytes. *Trends Immunol.* 23 549–555. 10.1016/S1471-4906(02)02302-5 12401408

[B63] Marie-Egyptienne D. T. Lohse I. Hill R. P. (2013). Cancer stem cells, the epithelial to mesenchymal transition (EMT) and radioresistance: potential role of hypoxia. *Cancer Lett.* 341 63–72. 10.1016/j.canlet.2012.11.019 23200673

[B64] Martin B. J. (2013). Inhibiting vasculogenesis after radiation: a new paradigm to improve local control by radiotherapy. *Semin. Radiat. Oncol.* 23 281–287. 10.1016/j.semradonc.2013.05.002 24012342 PMC3768004

[B65] Martinive P. Defresne F. Bouzin C. Saliez J. Lair F. Grégoire V. (2006). Preconditioning of the tumor vasculature and tumor cells by intermittent hypoxia: implications for anticancer therapies. *Cancer Res.* 66 11736–11744. 10.1158/0008-5472.CAN-06-2056 17178869

[B66] Martinive P. Defresne F. Quaghebeur E. Daneau G. Crokart N. Grégoire V. (2009). Impact of cyclic hypoxia on HIF-1alpha regulation in endothelial cells–new insights for anti-tumor treatments. *Febs J.* 276 509–518. 10.1111/j.1742-4658.2008.06798.x 19077164

[B67] Merad M. Manz M. G. Karsunky H. Wagers A. Peters W. Charo I. (2002). Langerhans cells renew in the skin throughout life under steady-state conditions. *Nat. Immunol.* 3 1135–1141. 10.1038/ni852 12415265 PMC4727838

[B68] Mihaescu A. Thornberg C. Mattsson S. Wang Y. Jeppsson B. Thorlacius H. (2007). Critical role of P-selectin and lymphocyte function antigen-1 in radiation-induced leukocyte-endothelial cell interactions in the colon. *Dis. Colon Rectum.* 50 2194–2202. 10.1007/s10350-007-9065-7 17851717

[B69] Moeller B. J. Cao Y. Li C. Y. Dewhirst M. W. (2004). Radiation activates HIF-1 to regulate vascular radiosensitivity in tumors: role of reoxygenation, free radicals, and stress granules. *Cancer Cell* 5 429–441. 10.1016/S1535-6108(04)00115-1 15144951

[B70] Moeller B. J. Dreher M. R. Rabbani Z. N. Schroeder T. Cao Y. Li C. Y. (2005). Pleiotropic effects of HIF-1 blockade on tumor radiosensitivity. *Cancer Cell* 8 99–110. 10.1016/j.ccr.2005.06.016 16098463

[B71] Movahedi K. Laoui D. Gysemans C. Baeten M. Stangé G. Van den Bossche J. (2010). Different tumor microenvironments contain functionally distinct subsets of macrophages derived from Ly6C(high) monocytes. *Cancer Res.* 70 5728–5739. 10.1158/0008-5472.CAN-09-4672 20570887

[B72] Özdemir B. C. Pentcheva-Hoang T. Carstens J. L. Zheng X. Wu C. C. Simpson T. R. (2014). Depletion of carcinoma-associated fibroblasts and fibrosis induces immunosuppression and accelerates pancreas cancer with reduced survival. *Cancer Cell* 25 719–734. 10.1016/j.ccr.2014.04.005 24856586 PMC4180632

[B73] Pajonk F. Vlashi E. McBride W. H. (2010). Radiation resistance of cancer stem cells: the 4 R’s of radiobiology revisited. *Stem cells (Dayton Ohio)* 28 639–648. 10.1002/stem.318 PMC2940232 20135685

[B74] Paquette B. Baptiste C. Therriault H. Arguin G. Plouffe B. Lemay R. (2007). In vitro irradiation of basement membrane enhances the invasiveness of breast cancer cells. *Br. J. Cancer* 97 1505–1512. 10.1038/sj.bjc.6604072 17987037 PMC2360264

[B75] Park H. J. Griffin R. J. Hui S. Levitt S. H. Song C. W. (2012). Radiation-induced vascular damage in tumors: implications of vascular damage in ablative hypofractionated radiotherapy (SBRT and SRS). *Radiat. Res.* 177 311–327. 10.1667/RR2773.1 22229487

[B76] Pastouret F. Lievens P. Leduc O. Bourgeois P. Tournel K. Lamote J. (2014). Short time effects of radiotherapy on lymphatic vessels and restorative lymphatic pathways: experimental approaches ina mouse model. *Lymphology* 47 92–100. 25282875

[B77] Price J. G. Idoyaga J. Salmon H. Hogstad B. Bigarella C. L. Ghaffari S. (2015). CDKN1A regulates Langerhans cell survival and promotes Treg cell generation upon exposure to ionizing irradiation. *Nat. Immunol.* 16 1060–1068. 10.1038/ni.3270 26343536 PMC4620552

[B78] Qian L. W. Mizumoto K. Urashima T. Nagai E. Maehara N. Sato N. (2002). Radiation-induced increase in invasive potential of human pancreatic cancer cells and its blockade by a matrix metalloproteinase inhibitor. CGS27023. *Clin. Cancer Res.* 8 1223–1227. 11948136

[B79] Rao S. S. Thompson C. Cheng J. Haimovitz-Friedman A. Powell S. N. Fuks Z. (2014). Axitinib sensitization of high Single Dose Radiotherapy. *Radiother. Oncol.* 111 88–93. 10.1016/j.radonc.2014.02.010 24794795 PMC4278650

[B80] Rekers N. H. Zegers C. M. Yaromina A. Lieuwes N. G. Biemans R. Senden-Gijsbers B. L. (2015). Combination of radiotherapy with the immunocytokine L19-IL2: additive effect in a NK cell dependent tumour model. *Radiother. Oncol.* 116 438–442. 10.1016/j.radonc.2015.06.019 26138057

[B81] Ribatti D. Mangialardi G. Vacca A. (2006). Stephen Paget and the “seed and soil” theory of metastatic dissemination. *Clin. Exp. Med.* 6 145–149. 10.1007/s10238-006-0117-4 17191105

[B82] Rodel F. Frey B. Manda K. Hildebrandt G. Hehlgans S. Keilholz L. (2012). Immunomodulatory properties and molecular effects in inflammatory diseases of low-dose x-irradiation. *Front. Oncol.* 2:120 10.3389/fonc.2012.00120 PMC3457026 23057008

[B83] Russell N. S. Floot B. van Werkhoven E. Schriemer M. de Jong-Korlaar R. Woerdeman L. A. (2015). Blood and lymphatic microvessel damage in irradiated human skin: the role of TGF-β, endoglin and macrophages. *Radiother. Oncol.* 116 455–461. 10.1016/j.radonc.2015.08.024 26347496

[B84] Shan Y. X. Jin S. Z. Liu X. D. Liu Y. Liu S. Z. (2007). Ionizing radiation stimulates secretion of pro-inflammatory cytokines: dose-response relationship, mechanisms and implications. *Radiat. Environ. Biophys.* 46 21–29. 10.1007/s00411-006-0076-x 17072632

[B85] Shen C. J. Sharma A. Vuong D. V. Erler J. T. Pruschy M. Broggini-Tenzer A. (2014). Ionizing radiation induces tumor cell lysyl oxidase secretion. *BMC Cancer* 14:1471 10.1186/1471-2407-14-532 PMC4223762 25052686

[B86] Sofia Vala I. Martins L. R. Imaizumi N. Nunes R. J. Rino J. Kuonen F. (2010). Low doses of ionizing radiation promote tumor growth and metastasis by enhancing angiogenesis. *PLoS ONE* 5:e11222 10.1371/journal.pone.0011222 PMC2888592 20574535

[B87] Song C. W. Lee Y. J. Griffin R. J. Park I. Koonce N. A. Hui S. (2015). Indirect tumor cell death after high-dose hypofractionated irradiation: implications for stereotactic body radiation therapy and stereotactic radiation surgery. *Int. J. Radiat. Oncol. Biol. Phys.* 93 166–172. 10.1016/j.ijrobp.2015.05.016 26279032 PMC4729457

[B88] Sonveaux P. Brouet A. Havaux X. Grégoire V. Dessy C. Balligand J. L. (2003). Irradiation-induced angiogenesis through the Up-regulation of the nitric oxide pathway. *Cancer Res.* 63 1012–1019. 12615716

[B89] Sonveaux P. Dessy C. Brouet A. Jordan B. F. Grégoire V. Gallez B. (2002). Modulation of the tumor vasculature functionality by ionizing radiation accounts for tumor radiosensitization and promotes gene delivery. *FASEB J.* 16 1979–1981. 10.1096/fj.02-0487fje 12397083

[B90] Sonveaux P. Frérart F. Bouzin C. Brouet A. Dewever J. Jordan B. F. (2007). Irradiation promotes Akt-targeting therapeutic gene delivery to the tumor vasculature. *Int. J. Radiat. Oncol. Biol. Phys.* 67 1155–1162. 10.1016/j.ijrobp.2006.11.031 17276618

[B91] Speake W. J. Dean R. A. Kumar A. Morris T. M. Scholefield J. H. Watson S. A. (2005). Radiation induced MMP expression from rectal cancer is short lived but contributes to in vitro invasion. *Eur. J. Surgical Oncol.* 31 869–874. 10.1016/j.ejso.2005.05.016 16081236

[B92] Straussman R. Morikawa T. Shee K. Barzily-Rokni M. Qian Z. R. Du J. (2012). Tumour micro-environment elicits innate resistance to RAF inhibitors through HGF secretion. *Nature* 487 500–504. 10.1038/nature11183 22763439 PMC3711467

[B93] Surace L. Guckenberger M. van den Broek M. (2015). Radiation holidays stimulate tumor immunity. *Oncotarget* 6 15716–15717. 10.18632/oncotarget.4608 26158547 PMC4599217

[B94] Tachiiri S. Katagiri T. Tsunoda T. Oya N. Hiraoka M. Nakamura Y. (2006). Analysis of gene-expression profiles after gamma irradiation of normal human fibroblasts. *Int. J. Radiat. Oncol. Biol. Phys.* 64 272–279. 10.1016/j.ijrobp.2005.08.030 16257130

[B95] Takeda N. O’Dea E. L. Doedens A. Kim J. W. Weidemann A. Stockmann C. (2010). Differential activation and antagonistic function of HIF-{alpha} isoforms in macrophages are essential for NO homeostasis. *Genes Dev.* 24 491–501. 10.1101/gad.1881410 20194441 PMC2827844

[B96] Tsai C. S. Chen F. H. Wang C. C. Huang H. L. Jung S. M. Wu C. J. (2007). Macrophages from irradiated tumors express higher levels of iNOS, arginase-I and COX-2, and promote tumor growth. *Int. J. Radiat. Oncol. Biol. Phys.* 68 499–507. 10.1016/j.ijrobp.2007.01.041 17398016

[B97] Tsujino T. Seshimo I. Yamamoto H. Ngan C. Y. Ezumi K. Takemasa I. (2007). Stromal myofibroblasts predict disease recurrence for colorectal cancer. *Clin. Cancer Res.* 13 2082–2090. 10.1158/1078-0432.CCR-06-2191 17404090

[B98] Vacchelli E. Semeraro M. Enot D. P. Chaba K. Poirier Colame V. Dartigues P. (2015). Negative prognostic impact of regulatory T cell infiltration in surgically resected esophageal cancer post-radiochemotherapy. *Oncotarget* 6 20840–20850. 10.18632/oncotarget.4428 26369701 PMC4673233

[B99] Valerie K. Yacoub A. Hagan M. P. Curiel D. T. Fisher P. B. Grant S. (2007). Radiation-induced cell signaling: inside-out and outside-in. *Mol. Cancer Ther.* 6 789–801. 10.1158/1535-7163.MCT-06-0596 17363476

[B100] Vatner R. E. Formenti S. C. (2015). Myeloid-derived cells in tumors: effects of radiation. *Semi. Radiat. Oncol.* 25 18–27. 10.1016/j.semradonc.2014.07.008 25481262

[B101] Verset L. Tommelein J. Moles Lopez X. Decaestecker C. Boterberg T. De Vlieghere E. (2015). Impact of neoadjuvant therapy on cancer-associated fibroblasts in rectal cancer. *Radiother. Oncol.* 116 449–454. 10.1016/j.radonc.2015.05.007 26021554

[B102] Wagner-Ecker M. Schwager C. Wirkner U. Abdollahi A. Huber P. E. (2010). MicroRNA expression after ionizing radiation in human endothelial cells. *Radiat. Oncol.* 5 5–25. 10.1186/1748-717X-5-25 20346162 PMC2859352

[B103] Weigert A. Brune B. (2008). Nitric oxide, apoptosis and macrophage polarization during tumor progression. *Nitric Oxide* 19 95–102. 10.1016/j.niox.2008.04.021 18486631

[B104] Wu W. S. (2006). The signaling mechanism of ROS in tumor progression. *Cancer Metast. Rev.* 25 695–705. 10.1007/s10555-006-9037-8 17160708

[B105] Wu Z.-H. Miyamoto S. (2007). Many faces of NF-κB signaling induced by genotoxic stress. *J. Mol. Med.* 85 1187–1202. 10.1007/s00109-007-0227-9 17607554

[B106] Xu J. Escamilla J. Mok S. David J. Priceman S. West B. (2013). CSF1R signaling blockade stanches tumor-infiltrating myeloid cells and improves the efficacy of radiotherapy in prostate cancer. *Cancer Res.* 73 2782–2794. 10.1158/0008-5472.CAN-12-3981 23418320 PMC4097014

[B107] Yamashita M. Ogawa T. Zhang X. Hanamura N. Kashikura Y. Takamura M. (2012). Role of stromal myofibroblasts in invasive breast cancer: stromal expression of alpha-smooth muscle actin correlates with worse clinical outcome. *Breast Cancer* 19 170–176. 10.1007/s12282-010-0234-5 20978953

[B108] Yu H. Mohan S. Natarajan M. (2012). Radiation-triggered NF-kappaB activation is responsible for the angiogenic signaling pathway and neovascularization for breast cancer cell proliferation and growth. *Breast Cancer* 6 125–135. 10.4137/BCBCR.S9592 22872788 PMC3411495

[B109] Zong Z. W. Cheng T. M. Su Y. P. Ran X. Z. Shen Y. Li N. (2008). Recruitment of transplanted dermal multipotent stem cells to sites of injury in rats with combined radiation and wound injury by interaction of SDF-1 and CXCR4. *Radiat. Res.* 170 444–450. 10.1667/RR0744.1 19024651

